# Macrophage polarization as a potential therapeutic target for atherosclerosis: a dynamic stochastic modelling study

**DOI:** 10.1098/rsos.220239

**Published:** 2022-08-03

**Authors:** Mengchen Liu, Yan Cai, Jichao Pan, Karlheinz Peter, Zhiyong Li

**Affiliations:** ^1^ School of Biological Science and Medical Engineering, Southeast University, Nanjing 210096, People's Republic of China; ^2^ School of Mechanical, Medical and Process Engineering, Queensland University of Technology (QUT), Brisbane, QLD 4001, Australia; ^3^ Atherothrombosis and Vascular Biology, Baker Heart and Diabetes Institute, PO Box 6492, St Kilda Road Central, Melbourne, VIC 8008, Australia

**Keywords:** macrophages, polarization, target therapy

## Abstract

We proposed a dynamic stochastic mathematical model to evaluate the role of macrophage polarization in plaque development. The dynamic process of macrophages from proliferation to death was simulated under different lipid microenvironments. The probability of macrophage phenotypic switching was described using a Bernoulli distribution where the stochastic variable was determined by the local lipid level. Moreover, the interactions between macrophages and microenvironmental factors vary with macrophage phenotype. We investigated the distribution of key microenvironmental factors, the dynamics of macrophage polarization and its influence on foam cell formation. M1 macrophages were found to predominate in advanced plaque corresponding to the exacerbated inflammation observed in mice experiments. The imbalance between the deposition of oxidized low-density lipoprotein and phagocytic effects of macrophages governed the formation of foam cells. Furthermore, we simulated targeted therapies by either directly inhibiting the polarization probability to M1 macrophages or indirectly regulating macrophage polarization due to high-density lipoprotein levels. Comparison of simulation results with experimental findings in both therapies indicated that the intervention and regulation of macrophage polarization could influence plaque microenvironment and subsequently induce plaque regression, especially in the early stage. The proposed modelling system can facilitate the evaluation of novel therapies targeting macrophage polarization.

## 1. Introduction

Atherosclerosis is a chronic inflammatory disease, whose initiation and progression depend on local inflammation and accumulation of lipids in the artery wall [1]. Although many cells are involved in the development of atherosclerosis, including smooth muscle cells (SMCs), monocytes and foam cells, macrophages are acknowledged to be fundamental contributors. In atherosclerotic plaques, macrophages are submitted to a large variety of microenvironmental factors, such as cytokines and oxidized low-density lipoprotein (ox-LDL), which influence the phenotypic polarization and activation of macrophages, resulting in a dynamic plasticity. In experimental systems, the classical inflammatory macrophage phenotype has been termed M1 macrophages which are induced by T-helper 1 (Th-1). M1 macrophages contribute to a strong inflammatory programme, producing pro-inflammatory cytokines and chemokines, such as interleukin-1*β* (IL-1*β*), tumour necrosis factor-α and monocyte chemoattractant protein-1 (MCP-1), to markedly promote inflammation and kill pathogens. Alternatively, M2 macrophages induced by Th-2 cytokines have also been identified *in vitro*. M2 macrophages can produce anti-inflammatory factors, such as IL-10 and transforming growth factor-*β* (TGF-*β*), to improve tissue remodelling and repair by effective clearance of dying cells and debris [2–5]. Despite the extensive use of lipid-lowering therapies, such as statins to prevent progression of atherosclerosis, atherosclerotic cardiovascular disease remains the leading cause of mortality worldwide [6]. Since macrophage polarization has been demonstrated to play an important role in the progression of atherosclerotic plaques, promoting macrophage polarization to M2 phenotype would be a promising strategy for anti-atherosclerotic treatment. In fact, two main signalling pathways of M2 polarization (the JAK-STAT pathway and the Akt-p18-mTOR-LXR pathway) have been studied, which in fact provide several potential targets. Besides, several compounds and drugs are found to moderate M2 polarization in animal experiments [7].

Macrophages in atherosclerotic plaques are primarily derived from differentiated monocytes and self-proliferation, while they show various phenotypic transformation and functions depending on the density of lipoprotein in the microenvironment [8]. Macrophages tend to play a pro-inflammatory role in the region with a higher ox-LDL concentration, resulting in an increased probability for phenotypic switching to M1 macrophages. On the contrary, macrophages are more likely to be polarized to anti-inflammatory phenotype, i.e. M2 macrophages, in the region with a lower lipoprotein level [9,10]. Besides the lipid microenvironment, the intraplaque matrix and its mechanobiology affect the phenotypic switching of macrophages. Collagen concentration has been found to regulate macrophage phenotype and function, with progressive plaques with lower collagen content containing more pro-inflammatory macrophages, while more anti-inflammatory macrophages were found in declining plaques with higher collagen content [11,12]. The collagen matrix stimulates monocyte differentiation, promotes the uptake of LDL by macrophages [13,14] and alters the level of matrix metalloproteinase (MMP) secretion by macrophages. Lipid loading of SMCs impairs their ability to assemble fibrous extracellular matrix (ECM) and contributes to plaque instability [15]. Nevertheless, the characteristics of macrophage phenotypes in response to plaque microenvironmental signals *in vivo* are still unknown, and more work is needed to further develop new clinically translatable and practical therapeutic strategies [16].

The most important problem is that the M1 and M2 classification of macrophage phenotypes is based on *in vitro* experimental systems with unknown relevance to *in vivo* situation. Moreover, pure M1 and M2 macrophages almost certainly do not exist in atherosclerotic lesions. It is now understood that the phenotypic polarization of macrophages is a dynamic process which changes over time, since the nature and intensity of microenvironmental signals vary continuously during plaque progression [4,9]. Despite numerous experimental studies, mathematical models for describing the dynamic processes in atherosclerosis are rare. In addition, macrophage polarization was excluded in most of these models. In some pioneering modelling work for the reversal of cholesterol transport (RCT) during atherosclerosis, M1 and M2 macrophages were modelled by using partial differential equations (PDEs) to describe the effect of efferocytosis on plaque progression. It was found that the increase in the proportion of M2 macrophages after RCT was more likely to promote plaque regression [17–20]. However, none of these models has considered the dynamic transformation between different phenotypes of macrophages and their interactions with other microenvironmental components of the plaque. A novel mathematical modelling system is needed to quantify the dynamic pathophysiological processes of macrophage polarization during plaque development.

In a previous study, we proposed a multi-physical mathematical model by coupling lipid deposition, monocyte/macrophage recruitment and intraplaque angiogenesis to investigate the dynamic evolutions of plaque development in response to the varying local microenvironment [21–23]. A series of coupled reaction–diffusion equations were set up to describe the spatio-temporal changes of the main cellular and acellular components within the growing plaque, including macrophage recruitment, efferocytosis and apoptosis. However, the polarization of macrophages and its dynamic interactions with microenvironmental factors could not be well described by the traditional reaction–diffusion equations. To this end, a dynamic stochastic modelling system is established in this study by considering the following three main assumptions: (i) The probability of macrophage phenotypic switching to M1/M2 is assumed to be stochastic variables with the Bernoulli distribution. (ii) The parameter *p* in the Bernoulli distribution is determined by the local lipid environment. (iii) The parameters denoting the interactions among macrophages and microenvironmental factors are assumed to change with the varying phenotype of macrophages. In this context, we investigate the distribution of key microenvironmental factors, including ox-LDL, macrophages and foam cells, during different stages of plaque progression (early, baseline and advanced). Additionally, we analyse the dynamics of macrophage polarization at different stages and its influence on foam cell formation. Moreover, we simulate the changes of plaque microenvironment in targeted therapies and compare the simulation results with animal experimental findings quantitatively. Finally, we discuss potential mechanisms of the effects of high-density lipoprotein (HDL) on plaque stabilization due to the regulation of macrophage polarization.

## 2. Methods

### 2.1. Study design and basic model settings

**Figure 1.**
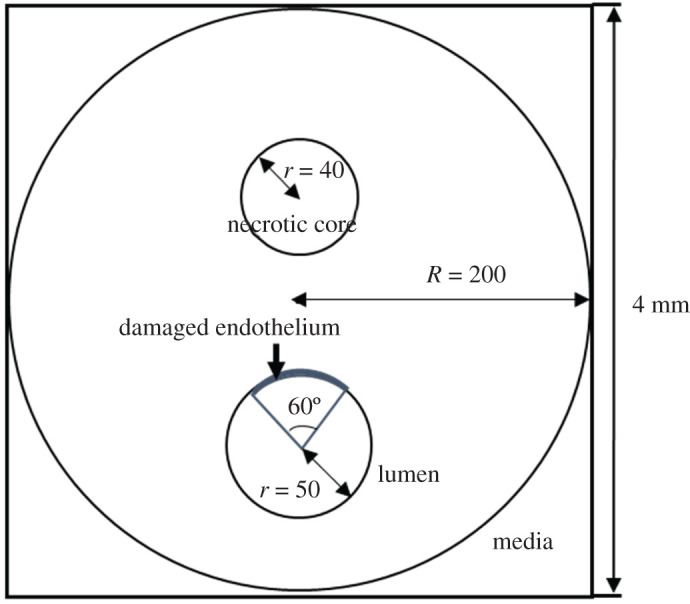
Schematic diagram of the model simulation area.

The mathematical model was established on a two-dimensional square area (4 mm × 4 mm), dividing into 400 × 400 grids equally. We set the region of arterial media as a circle with a radius of 200 grids and the centre located at (200, 200). The lumen centre was located at (200, 250) and its radius was 50 grids. The necrotic core (NC) was set to be a circular region with radius of 40 grids, whose centre aligned with the centre of the lumen (figure 1). The upper part of the lumen (corresponding to a circular angle of 60°) was considered as the region with dysfunctional endothelium.

**Figure 2.**
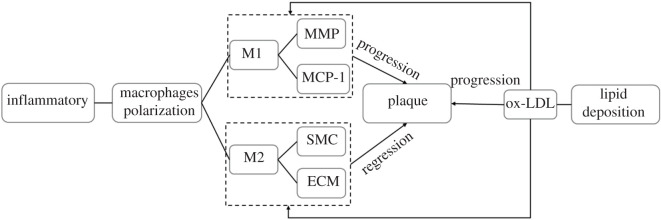
Schematic diagram of the interactions of the main factors in the model.

Figure 2 shows the interactions among the key factors involved in the model. Generally, the lipid microenvironment regulates the macrophage polarization and consequently influences the plaque progression due to the dynamic inflammatory environment and its impact on other components within the plaque. In particular, M1 macrophages have been found to secrete pro-inflammatory mediators including MMPs and MCP-1, leading to the reduction of cholesterol efflux and the degradation of ECM. M2 macrophages secrete anti-inflammatory cytokines, such as TGF-*β*, IL-1 receptor antagonist, IL-10, promoting ECM synthesis by increasing collagen content and enhancing capacity of efferocytosis which contribute to the regression of plaque inflammation [24,25]. The bidirectional regulation of lipid deposition on macrophage phenotype was modelled by a probability density function, which is illustrated in detail in §2.2.

Based on our previous work [21,22], we developed a coupled modelling system of dynamics of microenvironmental factors involved in plaque progression. The pathophysiological phenomena considered in the model included lipid deposition, inflammation, SMC proliferation and intraplaque angiogenesis. We assumed nine microenvironmental components (LDL, HDL, ox-LDL, macrophages, monocytes, ECM, MCP-1, MMP and SMCs) to be functions of space and time, which were described by a system of coupled PDEs. In addition, the interaction coefficients of macrophages with other factors were assumed to be dynamically changeable according to local lipid microenvironment. The detailed descriptions and explanations of the coupled PDEs can be found in §2.3.

All chemicals and cells remained within the domain during the simulations and no-flux boundary condition was applied. All components were normalized into a range of 0 to 1. The reference values for normalization of each variable are listed in table 1. Wherever possible, the reference values have been estimated from available experimental data. For example, the physiological range for LDL is 70–130 mg dl^−1^ and for HDL is 45–60 mg dl^−1^, both *L* and *H* are estimated here as 7 × 10^−4^ g cm^−3^ for ease of calculation. ox-LDL is estimated to be 2 × 10^−7^ g cm^−3^ to characterize lipid deposition in the baseline plaque. Monocyte density *Mo* is 5 × 10^−5^ g cm^−1^ (range from 20 000 to 100 000 cells per ml); macrophages convert from monocytes so macrophage density *M* is estimated to be 4 × 10^−5^ g cm^−3^. Previous model [18] provided empirical values, including the concentrations of MMP (3 × 10^−8^ g cm^−3^), MCP-1 (3 × 10^−10^ g cm^−3^) and SMCs (6 × 10^−3^ g cm^−3^). Researchers [26] found that ECM (up to 8 mg ml^−1^) promotes a significant acute endogenous repair response, so we set 4 × 10^−2^ g cm^−3^ as the value of ECM.

**Table 1.** The variables involved in the model and their baseline values.

parameter	description	baseline value (g cm^−3^)
ECM	density of ECM	4 × 10^−2^ [22]
*L*	concentration of LDL	7 × 10^−4^ [18]
*H*	concentration of HDL	7 × 10^−4^ [18]
*L*_ox_	concentration of ox-LDL	2 × 10^−7^^a^
MCP	concentration of MCP-1	3 × 10^−10^ [18]
MMP	concentration of MMPs	3 × 10^−8^ [22]
SMC	density of SMCs	6 × 10^−3^ [22]
*Mo*	density of monocytes	5 × 10^−5^ [22]
*M*	density of macrophages	4 × 10^−5^ [22]
*F*	density of foam cells	2 × 10^−8^^a^

^a^Estimated.

The initial densities of the cells as well as the initial distributions of chemicals were defined as dimensionless constants. In particular, we assumed three conditions to represent three stages during plaque development, i.e. early, baseline and advanced plaque. In the baseline model, the initial distributions of LDL, HDL, monocytes and macrophages were assumed to be accumulated in the plaque area with a small amount of ox-LDL deposited in the NC region. In the early model, no ox-LDL was assumed, and the lipoproteins and inflammatory cell concentrations were half of those in the baseline model; while in the advanced model, the lipoproteins and inflammatory cells were assumed to be two times the baseline level. Other microenvironmental factors (SMCs, MCP-1, MMP and ECM) were assumed to be distributed in the annulus between the intima and the media uniformly in all three stage models. The detailed settings of the microenvironmental factors for each stage are listed in table 2. Besides, the intraplaque neovasculature was represented by randomly distributed endothelial cells (ECs) with different densities in the simulation region. The density of the ECs, i.e. the neovascularization, varied in different stages of plaque development. The source of LDL and monocytes within the plaque included not only the influx from the damaged intima but also the extravasation from the neovascularization.

**Table 2.** Three microenvironmental conditions correspond to different stages of plaque progression, i.e. the early, baseline and advanced stages. All values are non-dimensional.

	ox-LDL	LDL	HDL	monocytes	macrophages	EC density
early	0	0.5	0.5	0.5	0.5	0.05
baseline	0.05	1	1	1	1	0.10
advanced	0.2	2	2	2	2	0.20

### 2.2. Bernoulli distribution for macrophage polarization

In this study, both the probability of macrophage polarization and the coefficients of interactions of macrophages with other factors were assumed to be associated with the ox-LDL concentration in the plaque lesion. In particular, when the lipid density in the plaque was moderate, the probability of macrophage polarization to M1 or M2 was equal. However, if the intraplaque lipoprotein level deviated from the average range, the probability of polarization would be biased accordingly.

Based on the above basic understanding, we used Bernoulli distribution with parameter *p* to describe the stochastic model of macrophage polarization at different lipid level. Specifically, the stochastic variable *x* was assumed to be 1 when macrophages transferred to M1 macrophages, while *x* = 0 for M2 macrophages. The probability of polarization to M1 macrophages (*p*) was assumed to be different constants in response to different levels of ox-LDL concentration (equation (2.1)). Accordingly, the probability of polarization to M2 macrophages was 1 − *p*. We divided the concentration of ox-LDL into high, medium and low levels, based on the comparison with the maximum value (*θ*_max_) of ox-LDL throughout the plaque region. The probability function of *x* was as follows:

$$f(x|p)=\{\begin{array}{ll} \;p^{x}{\left(1-p\right)}^{1-x},\; & x=0,1\;\\ 0,\; & x\neq 0,1 \end{array}$$

2.1 $$p=\{\begin{array}{ll} 0.8, & \mathrm{ox}\text{-}{\mathrm{LDL}}_{ij}>0.001\;{}^{*}\;\theta_{\max}\\ 0.5, & 0.000001\;{}^{*}\;\theta_{\max}<\mathrm{ox}\text{-}{\mathrm{LDL}}_{ij}\leq 0.001\;{}^{*}\;\theta_{\max}\\ 0.2, & \mathrm{ox}\text{-}{\mathrm{LDL}}_{ij}\leq 0.000001\;{}^{*}\;\theta_{\max}. \end{array}$$

### 2.3. Evolution of microenvironment

All microenvironmental components and their interactions among them were modelled by a system of PDEs based on our previous work [21,22]. In each evolution period (*T*), 10 PDEs were solved simultaneously to obtain the evolution of cellular and acellular components within the plaque (equations (2.2)–(2.11)).

#### 2.3.1. Lipid deposition

2.2 $$\frac{\partial L}{\partial t}=D_{L}\nabla^{2}L-\lambda_{L}\cdot L,$$

2.3 $$\frac{\partial H}{\partial t}=D_{H}\nabla^{2}H-d_{H}H,$$

2.4 $$\frac{\partial L_{\mathrm{ox}}}{\partial t}=D_{L_{\mathrm{ox}}}\nabla^{2}L_{\mathrm{ox}}+\lambda_{L_{\mathrm{ox}}}L-\lambda_{M\cdot L_{\mathrm{ox}}}\frac{L_{\mathrm{ox}}}{K_{L_{\mathrm{ox}}}+L_{\mathrm{ox}}}M$$

2.5 $$\;\mathrm{and}\;\frac{\partial F}{\partial t}=D_{F}\nabla^{2}F+\lambda_{M\cdot L_{\mathrm{ox}}}\frac{L_{\mathrm{ox}}}{K_{L_{\mathrm{ox}}}+L_{\mathrm{ox}}}M-d_{F}F.$$

Equations (2.2) and (2.3) describe the dynamics of LDL and HDL, respectively. The first term on the right is the diffusion of LDL or HDL within the plaque with diffusion coefficient of $D_{L}$ or $D_{H}$ and the second term is the decay of themselves. ox-LDL is derived from the oxidation of LDL as shown in the second term on the right of equation (2.4) and absorbed by macrophages with a coefficient of $\lambda_{M\cdot L_{\mathrm{ox}}}$. The origin of foam cells is thought to be the result of macrophage engulfment of a large amount of ox-LDL overload (second term on the right-hand side of equation (2.5)).

#### 2.3.2. Inflammatory cells

2.6 $$\frac{\partial Mo}{\partial t}=D_{Mo}\nabla^{2}Mo-\nabla \cdot (\lambda_{Mo\cdot L_{\mathrm{ox}}}Mo\nabla L_{ox})-d_{Mo}Mo,$$

2.7 $$\begin{array}{c} \frac{\partial M}{\partial t}=D_{M}\nabla^{2}M+\lambda_{M}\cdot M_{0}-\lambda_{F\cdot M}\left(\frac{L_{ox}}{K_{L_{ox}}+L_{ox}}\right)M-d_{M}M-\nabla (\lambda_{MCP\cdot M}M\nabla MCP) \end{array}$$

2.8 $$\;\mathrm{and}\;\frac{\partial MCP}{\partial t}=D_{MCP}\nabla^{2}MCP+\lambda_{S\cdot MCP}S+\lambda_{M\cdot MCP}M-d_{MCP}MCP.$$

The first term on the right-hand side of equation (2.6) indicates monocyte diffusion and the last two terms describe the chemotaxis of monocytes in response to ox-LDL and their own decay. In equation (2.7), the second term on the right indicates the conversion of monocytes to macrophages, and the last term indicates the chemotaxis of macrophages in response to MCP-1. At initial stage, macrophages can remove lipoproteins and apoptotic cells by effective efferocytosis; however, overload of lipids converted macrophages into foam cells and eventually contributes to the formation of NC. The third term in equation (2.7) corresponds to this process and the coefficient $\lambda_{F\cdot M}$ varies with the local lipoprotein concentration. In response to lipid microenvironment, SMCs and macrophages secrete MCP-1 to enhance monocyte aggregation (equation (2.8), the second and third terms from the right).

#### 2.3.3. Matrix

2.9 $$\frac{\partial ECM}{\partial t}=-\lambda_{MMP\cdot ECM}ECM\cdot MMP+\lambda_{S\cdot \mathrm{ECM}}S+\lambda_{M\cdot ECM}M,$$

2.10 $$\begin{array}{c} \frac{\partial S}{\partial t}=D_{S}\nabla^{2}S-\nabla (\lambda_{MCP\cdot S}S\nabla MCP)-\nabla (\lambda_{M\cdot S}S\nabla M)-\nabla (\lambda_{ECM\cdot S}S\nabla ECM) \end{array}$$

2.11 $$\begin{array}{c} \;\mathrm{and}\;\frac{\partial MMP}{\partial t}=D_{\mathrm{MMP}\;}\nabla^{2}MMP+\lambda_{S\cdot MMP}S+\lambda_{M\cdot \mathrm{MMP}}M-d_{\mathrm{MMP}\;}MMP \end{array}.$$

ECM can be degraded by MMP and produced by SMCs and macrophages (equation (2.9)). Besides the chemotaxis in response of MCP-1 and macrophages, the movement of SMCs is also influenced by the hepatotactic effect of ECM (the last term in equation (2.10)). MMP can diffuse and be produced by SMCs and macrophages (equation (2.11)).

Since macrophages display remarkable heterogeneity as a result of different phenotypes and eventually contribute to plaque development towards diverse fate, the coefficients of the interaction of macrophages with other factors were assumed to change with the ox-LDL concentration in the plaque lesion (table 3) [27]. For example, macrophages are more likely to be M1 macrophages in high level of ox-LDL, contributing to the inflammatory microenvironment with increasing production of MCP-1 and MMP. On the contrary, M2 macrophages stimulated by low level of ox-LDL could enhance the secretion of ECM and VEGF by macrophages, suggesting the characteristics of advanced atherosclerotic lesions. We used the highest concentration of ox-LDL within the plaque (*θ*_max_) as the threshold and divided the calculated region into high, medium and low lipid regions. The ox-LDL concentration greater than one thousandth of *θ*_max_ was considered the high-lipid zone, and below 10^−6^*θ*_max_ was the low-fat zone, and the area where the ox-LDL concentration was between these two was in medium-lipid range.

**Table 3.** Different values of interaction coefficient of macrophages with other microenvironmental factors. All values are non-dimensional.

lipid range	$\lambda_{M\cdot ECM}$	$\lambda_{M\cdot MCP}$	$\lambda_{M\cdot MMP}$	$\lambda_{M\cdot SMC}$	$\lambda_{M\cdot Lox}$	$\lambda_{M\cdot F}$	$d_{M}$
low	5 × 10^−5^	3 × 10^−5^	3 × 10^−5^	5 × 10^−5^	3	0.5	2
medium	4 × 10^−5^	4 × 10^−5^	4 × 10^−5^	4 × 10^−5^	2	1	1
high	3 × 10^−5^	5 × 10^−5^	5 × 10^−5^	3 × 10^−5^	1	1.5	0.5

### 2.4. Simulation algorithm

**Figure 3.**
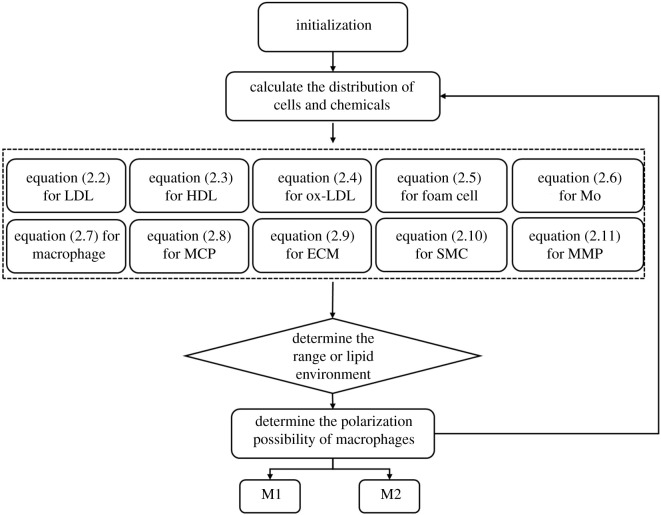
Schematic diagram of the coupled model simulation algorithm.

The coupled model consisted of two sub-loops (figure 3): (i) a system of PDEs for cellular and chemical changes in plaque and (ii) a probability density function for macrophage polarization. First, we generated a neovascular network through a stochastic model as a source of lipoproteins and monocytes; lipoproteins were then oxidized to ox-LDL, which upregulated the expression of pro-inflammatory cytokines such as MCP-1 and MMP, which in turn allowed more monocytes to enter the plaque and the original macrophages to start proliferating. After updating the concentrations of cellular and acellular components, macrophages polarized into different phenotypes with certain probability, generating feedback to the lipid microenvironment in the next coupling of sub-loops.

Equations (2.2)–(2.11) were normalized by rescaling the diameter distance of the carotid artery, time with *τ* = *L*^2^/*D_M_* (where *D_M_* is macrophages diffusion coefficient). Since the average diameter of the carotid artery is between 4 and 8 mm, the length scale *L* was considered as 4 mm. In order to satisfy the stability condition $|(\Delta t/\Delta x^{2})D_{M}|\leq 1/4$, we set a spatial step of 20 μm and a time step of 0.1 s. The reaction–diffusion simulations ended with a condition that the relative error was less than 10^−4^ after iterative calculations for each substance, and the overall simulation ended after 6000 steps of iterations.

Considering the stochastic effects in the modelling, ten simulations were performed for each microenvironment condition and the average results are shown in the following section. All parameters involved in the model are listed in table 4 with their normalized values.

**Table 4.** The description and normalized values of all parameters involved in the model.

parameter	description	value
$D_{L}$	diffusion coefficient of LDL	0.1 [28,29]
$D_{H}$	diffusion coefficient of HDL	0.1 [28,29]
$D_{Lox}$	diffusion coefficient of ox-LDL	0.08 [28,29]
$D_{F}$	diffusion coefficient of foam cells	0.08 [30,31]
$D_{Mo}$	diffusion coefficient of monocytes	0.08 [30,31]
$D_{M}$	diffusion coefficient of macrophages	0.08 [30,31]
$D_{MCP}$	diffusion coefficient of MCP-1	0.01 [32]
$D_{S}$	diffusion coefficient of SMCs	0.02 [30,31]
$D_{MMP}$	diffusion coefficient of MMP	0.2 [33]
$\lambda_{Lox}$	rate of reaction of LDL with free radicals to form ox-LDL	0.005 [18]
$\lambda_{M\cdot Lox}$	rate of ox-LDL ingestion by macrophages	0.01 [34]
$\lambda_{Mo\cdot Lox}$	rate of ox-LDL ingestion by monocytes	0.0001 [34]
$\lambda_{M}$	production rate of macrophages by monocytes	0.005 [35]
$\lambda_{F\cdot M}$	rate of cytokinesis of lipoproteins by macrophages	0.01 [35]
$\lambda_{MCP\cdot M}$	chemotaxis coefficient of macrophages to MCP-1	0.004 [18]
$\lambda_{S\cdot MCP}$	production rate of MCP-1 by SMCs	0.0001 [18]
$\lambda_{M\cdot MCP}$	production rate of MCP-1 by macrophages	0.0001 [18]
$\lambda_{MMP\cdot ECM}$	rate of ECM degradation due to MMP	0.00045 [18]
$\lambda_{S\cdot ECM}$	production rate of ECM by SMCs	0.001 [18]
$\lambda_{M\cdot ECM}$	production rate of ECM by macrophages	0.001 [18]
$\lambda_{MCP\cdot S}$	chemotaxis coefficient of SMCs to MCP-1	0.0006 [18]
$\lambda_{M\cdot S}$	chemotaxis coefficient of SMCs to macrophages	0.001 [18]
$\lambda_{ECM\cdot S}$	migration coefficient of SMCs due to ECM	0.001 [18]
$\lambda_{S\cdot MMP}$	production rate of MMP by SMCs	0.01 [30]
$\lambda_{M\cdot MMP}$	production rate of MMP by macrophages	0.01 [18]
$\lambda_{A\cdot MMP}$	production rate of MMP by ECs	0.075 [18]
$\lambda_{H\cdot F}$	rate of HDL transportation for oxidation of LDL	0.002 [18]
$d_{L}$	degradation rate of LDL	0.005 [18]
$d_{H}$	degradation rate of HDL	0.003 [18]
$d_{F}$	death rate of foam cells	0.0001 [35]
$d_{Mo}$	death rate of monocytes	0.005 [35]
$d_{M}$	death rate of macrophages	0.0001 [35]
$d_{MCP}$	degradation rate of MCP-1	0.0001 [32]
$d_{MMP}$	degradation rate of MMP	0.001 [29]
$K_{L_{ox}}$	ox-LDL saturation for production of MCP-1	0.5 [28]
$K_{H}$	HDL saturation for transportation of foam cells	0.5 [28]

## 3. Results

### 3.1. Distributions of microenvironmental factors

**Figure 4.**
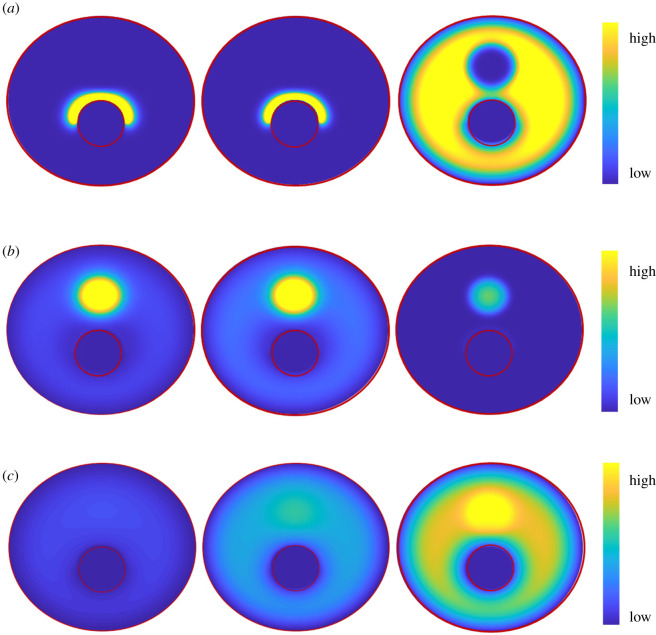
The distribution of ox-LDL (*a*), macrophages (*b*) and foam cells (*c*) within the plaque area in early, baseline and advanced models. The red circle indicates the lumen area.

The distributions of the main microenvironmental factors (ox-LDL, macrophages and foam cells) were investigated under different evolutional stages of plaque progression (figure 4). In the early and baseline models, the ox-LDL typically accumulated around the lumen boundary. The deposition of ox-LDL within the plaque further induced proliferation and migration of macrophages, to remove ox-LDL and form foam cells due to the phagocytosis. Few foam cells were found in the NC region corresponding to the distribution of high-level macrophages, suggesting a balance between deposition of ox-LDL and phagocytic effects. In the advanced model, however, the rapid aggregation of ox-LDL exceeded the phagocytic efficiency of macrophages, resulting in the exacerbated formation of foam cells around the NC area.

**Figure 5.**
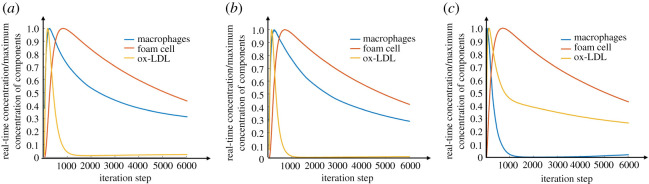
Changes of intraplaque macrophage, ox-LDL and foam cell contents over time in early (*a*), baseline (*b*) and advanced (*c*) models.

The quantitative comparison of these three microenvironmental factors at different stages can be found in the curves in figure 5. In the early and baseline models, the ox-LDL reached a peak value first, followed by the macrophages and foam cells. In the advanced model, however, a rapid reduction of macrophages was found before the decrease of ox-LDL. It was noteworthy that a high level of ox-LDL with a near-zero level of macrophages occurred in the advanced model, which suggested that the phagocytic efficiency of macrophages was deficient to eliminate the ox-LDL in the plaque. This imbalance contributed to the formation of foam cells in the advanced model as observed in figure 5*c*.

### 3.2. Dynamics of macrophage polarization

**Figure 6.**
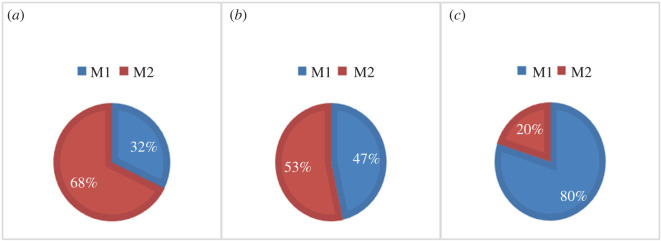
The proportion of macrophages polarized to M1 or M2 within the plaque in the early (*a*), baseline (*b*) and advanced (*c*) models.

Figure 6 shows the changes of proportion of M1/M2 macrophages in different microenvironmental conditions. Both the M1 and M2 phenotypes were found in the early, baseline and advanced lesions. However, during plaque progression, the number of M2 macrophages decreased while the number of M1 macrophages increased. Approximately 47% of macrophages were polarized to M1 type in the baseline model, while 32% to M1 macrophages in the early model and 80% in the advanced model. In parallel, M2 macrophages decreased from 68% in the early model to 20% in the advanced model.

**Figure 7.**
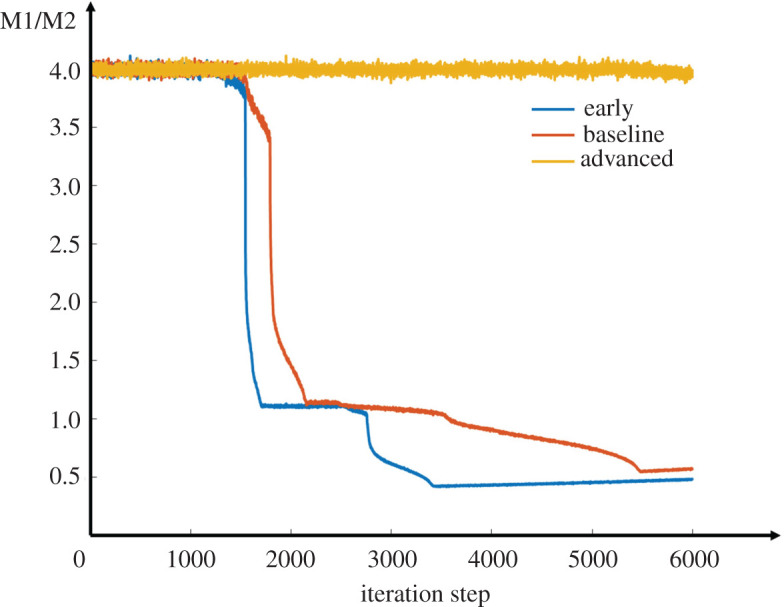
Changes of M1/M2 ratio in the three different models.

Next, we examined the ratio of M1 to M2 macrophages and its relationship with the lipid level in plaque. As shown in the curves in figure 7, with the same initial ratio of M1 to M2, the ratio decreased in the early and baseline models, while it barely changed in the advanced model. As a result, M1 macrophages dominated in advanced plaque, with a value almost four times that of M2 macrophages. This result indicated that the lipid microenvironment was maintained at a high level in the advanced model. In other words, there was no efficient clearance of lipoprotein by macrophages in the advanced model.

### 3.3. Effects of targeted therapy on plaque microenvironment

Since the dynamics of macrophages' polarization contribute to the formation of foam cells, one can deduce that inhibiting M1 polarization and/or promoting M2 polarization may provide a new approach to alleviate inflammation and increase plaque stability. In fact, *in vivo* experiments in mice have demonstrated that the anti-inflammatory agent ginsenoside Rb1 promoted M2 macrophage polarization and consequently enhanced plaque stability by increasing IL-4 and/or IL-13 production and STATG phosphorylation [36]. Here, we investigated the effect of Rb1 on plaque microenvironment by regulating macrophage polarization. In particular, we adjusted the probability of macrophage polarization in the model as follows and applied the updated probability function in early, baseline and advanced conditions, respectively:

$$f(x|p)=\{\begin{array}{ll} \;p^{x}{\left(1-p\right)}^{1-x},\; & x=0,1\;\\ 0,\; & x\neq 0,1\; \end{array}$$

3.1 $$p=\{\begin{array}{ll} 0.7, & \mathrm{ox}\text{-}{\mathrm{LDL}}_{ij}>0.001\;{}^{*}\;\theta_{\max}\\ 0.4, & 0.000001\;{}^{*}\;\theta_{\max}<\mathrm{ox}\text{-}{\mathrm{LDL}}_{ij}\leq 0.001\;{}^{*}\;\theta_{\max}\\ 0.1, & \mathrm{ox}\text{-}{\mathrm{LDL}}_{ij}\leq 0.000001\;{}^{*}\;\theta_{\max}. \end{array}$$

**Figure 8.**
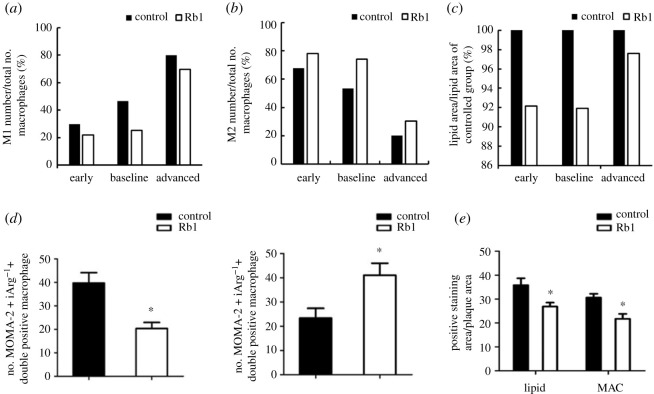
(*a*) Changes in M1 concentration in early, baseline and advanced stage models after using Rb1-targeted macrophage therapy. (*b*) Changes in M2 concentration in the three models after using Rb1-targeted macrophage therapy. (*c*) Rate of change in lipoprotein area in the three models after using Rb1-targeted macrophage therapy. (*d*,*e*) Cell staining results of *in vitro* sections of mouse plaques before and after treatment with Rb1 [36].

**Figure 9.**
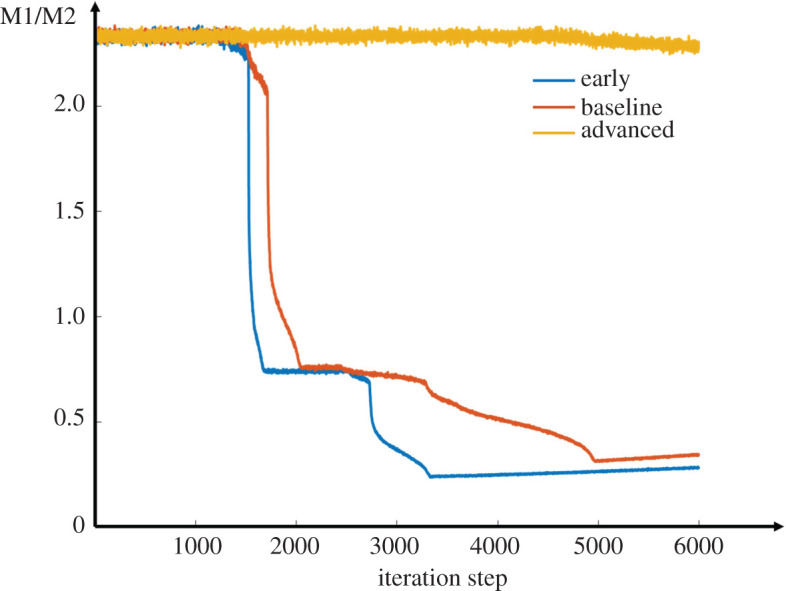
Changes of M1/M2 ratio after targeted therapy in early, baseline and advanced models.

The results (figure 8) demonstrate the efficacy of Rb1 on promoting macrophage polarization to M2 phenotype as well as on inhibiting M1 polarization. Consistent with the experimental results [36], under all three microenvironmental conditions, the reduction of M1 macrophages and the increase of M2 macrophages was about 14% after introduction of Rb1. In addition, the relative area of lipoprotein in the plaque decreased by about 8%. It was noteworthy that although the change in M1 reduction and M2 increase occurred, the M1 type was still predominant (70%) in macrophages observed in the advanced model after targeted therapy. Thus, the reduction of lipid area was less than 5% in the advanced model. By contrast, there were nearly 10% reductions of lipid area in the early and baseline models. We further investigated the curves of M1 to M2 ratio after Rb1 therapy (figure 9). Similar to the results in the non-therapy control models, the ratio of M1 to M2 decreased in both the early and baseline models, while it stayed around 2.5 in.the advanced model.

### 3.4. Effects of high-density lipoprotein on atherosclerosis by regulating macrophage polarization

Based on the above observations, we hypothesized that the efficient removal of lipoprotein could change the lipid microenvironment in the plaque and subsequently regulate the polarization of macrophages. Here, the effect of RCT caused by HDL cholesterol was included in the current model. Specifically, HDL transports cholesterol by RCT with a coefficient of $\lambda_{H\cdot F}$, as in the third term on the right-hand side in equation (3.2). For simplicity, the removal of foam cells due to the RCT was also modelled by the same term in equation (3.3).

3.2 $$\frac{\partial H}{\partial t}=D_{H}\nabla^{2}H-\lambda_{H}H-\lambda_{H\cdot F}\left(\frac{F}{K_{H}+F}\right)H$$

and

3.3 $$\frac{\partial F}{\partial t}=D_{F}\nabla^{2}F+\lambda_{M\cdot L_{\mathrm{ox}}}\frac{L_{\mathrm{ox}}}{K_{L_{\mathrm{ox}}}+L_{\mathrm{ox}}}M-\lambda_{H\cdot F}\frac{F}{K_{H}+F}H-d_{F}F.$$

**Figure 10.**
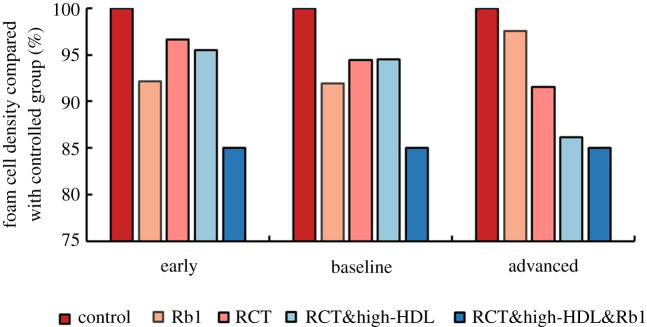
Effect of Rb1-targeted macrophages, addition of RCT cholesterol reversal transport effect, HDL-raising therapy, and combined HDL and Rb1 treatment on intraplaque foam cell concentration at early, baseline and late stages.

There are experimental results supporting that an elevated HDL level promoted plaque regression [37,38]. Therefore, we investigated the influence of a higher HDL level, i.e. increasing HDL levels by 50%, on the foam cell formation. We compared the effects of three kinds of therapies on foam cells, which were targeted therapy (Group Rb1), lipid-control therapy (Group RCT, Group RCT&high-HDL) and synergistic therapy (Group RCT&high-HDL&Rb1). As shown in figure 10, remarkable decreases of foam cells were found in all three models in Group RCT&high-HDL&Rb1 (15–17%). In the early and baseline model, Rb1-targeted therapy produced more reduction of foam cells than the lipid-control therapy. In the advanced model, however, the effect of lipid-control therapy was more significant than targeted therapy only.

## 4. Discussion

Although statins have been widely used for the treatment of atherosclerosis, more efficient novel drugs or adjuvant medicines are still needed for cardiovascular diseases. Growing evidence has demonstrated the important role of macrophage polarization in lesion development and plaque stability, suggesting that research on macrophage plasticity could lead to novel therapeutic approaches to counteract atherosclerosis. In order to investigate the dynamic phenotypic polarization of macrophages in response to lipid microenvironment, we introduced a probability density function in the coupled modelling system of plaque progression. In addition, the interactions between macrophages and other microenvironmental factors were investigated by changing the coefficients in the reaction–diffusion equations to implement the functional consequences of the phenotypic heterogeneity of macrophages. The dynamics of macrophage polarization under different lipid conditions were performed by using this dynamic stochastic model. The simulation results revealed the different characteristics of M1 and M2 macrophages in digesting lipoproteins and forming foam cells, which would contribute to determining their potential role in plaque development. Furthermore, we carried out simulations of targeted therapies by either directly inhibiting the probability of M1 macrophage polarization or indirectly the regulation of macrophage polarization due to HDL levels. The results in both therapies indicated that the intervention and regulation of macrophage polarization could influence plaque microenvironment and subsequently change the fate of the plaque, especially in the early stage of plaque progression. It was found that the plaque was more likely to develop to a stable lesion by upregulation of proportion of M2 macrophages and local HDL levels, due to the reduction of foam cells.

The main innovation of this study is the introduction of a probability density function to describe the dynamics of macrophage polarization during plaque development. Additionally, the probabilities for macrophages’ phenotypic switching were assumed to be associated with local ox-LDL concentration rather than pre-setting coefficients. Recent studies have demonstrated that lesional macrophages display remarkable heterogeneity as a result of effects of microenvironmental factors. Actually, neither M1 nor M2 macrophages are present in atherosclerotic plaques alone where macrophages are exposed to a complex microenvironment and respond with the expression of different cell-surface markers to exert diverse functions. In this context, the dynamic stochastic model proposed in this study can be used to simulate the changing phenotypes of macrophages during plaque progression. This is particularly useful in cases that the factors affecting the macrophage polarization, such as lipids, cytokines and growth factors, can be measured by experiments. In addition, the setting of the probability density function can be expanded by including more contributing factors and more macrophage phenotypes, since new phenotypes and mechanisms underlying macrophage polarization are discovered continuously with ongoing research.

Another innovation of the current model is the coefficients of the interactions of macrophages with microenvironmental factors that are assumed to be dynamically changing with the phenotypes of macrophages. The diverse functions of macrophages to plaque progression in response to different lipid microenvironment can be investigated by using this PDE system with varying coefficients. For example, the production of MCP-1 by macrophages was assumed to increase with lipid levels, since the high expression of M1 macrophages contributes to the inflammatory microenvironment in the plaque. Another example, the formation coefficient of foam cells caused by the efferocytosis of macrophages was set to be changeable according to the different phenotype. It is noteworthy that the interactions among these plaque microenvironmental factors are coupled and multidirectional. Although the effects of macrophages on microenvironmental factors may be explicit, the impacts of phenotypic switching at cell level and cell–cell interactions on plaque progression at tissue level are implicit and imperceptible. The simulation results further highlight the complexity and cross-talk of intercellular plaque microenvironmental factors. For instance, it was found that M1 macrophages were predominant in the advanced plaque model. Although M1 macrophages demonstrated more ability to efferocytosis, the deposition of foam cells was observed to be significant in the advanced plaque due to the accumulation of lipid as well as inflammatory cytokines by M1 macrophages. This result suggested that the imbalance between deposition of lipoproteins and phagocytic effects of macrophages rather than the quantity of lesional macrophages governs the fate of plaque development.

Further demonstration was made of this hypothesis through the investigation of the targeted therapy of macrophages. Two adjustments were carried out in this modelling study: one was the direct inhibition of M1 macrophages; the other was indirect mechanism by upregulation of HDL levels. The simulation results revealed that the reduction of foam cells was up to 5–10% if these interventions were applied at the early stage of atherosclerosis. This result is consistent with the experimental findings on animals [36]. However, the extent to which these mechanisms operate on human subjects is yet still unknown. In addition, the early stage model in this study corresponded to the low lipid environment. In other words, the reduction of foam cells due to the targeted therapy of macrophages is easier to be observed in a plaque with low lipid microenvironment. As predicted in figure 10, it is possible that therapeutic treatments targeting pro-atherogenic processes in macrophages can be additive or synergistic with lipid-lowering therapy in terms of cardiovascular risk reduction.

Another noteworthy result was the reduction of foam cells by increasing the HDL level. The main mechanism of the regulation of HDL on plaque stability is based on the effect of RCT. In particular, the resolving ability of macrophages on lipoprotein was enhanced by elevated HDL levels, resulting in the decrease of foam cells as shown in the simulation results. Unlike direct inhibitors of inflammatory cytokines or chemokines, pro-resolving mediators are less likely to compromise host defence. An experimental study in mice demonstrated the potential promise of pro-resolving therapy for atherosclerosis [39]. In addition, clinical trials have found that the atherosclerotic regression caused by statin benefits is partly derived from statin-induced increases in HDL [40,41]. Our simulation results are consistent with experimental and clinical findings, and more importantly, our model can demonstrate the quantitative and dynamic changes in their effects at different stages of plaque development.

Importantly, there are many ways to extend the applications of this mathematical model. For example, with more specific experimental data of targeted therapy, such as the quantitative measurement of targeted therapy on macrophage polarization, the present model can serve as a theoretical platform for evaluation and development of novel therapeutic strategies. Our model predicts that the intervention and regulation of macrophage polarization may have more efficiency on plaque stability in the early stage than in the advanced progression. Additional data, such as retrospective or prospective follow-up studies, are needed to test whether our findings can be generalized in clinical settings. Nonetheless, based on the comparison of different therapeutic strategies, we expect a more satisfying response to combinational therapy. From this point of view, an emerging research direction is to reveal the insight of the interplay between deposition of lipoproteins and phagocytic effects of macrophages on the ground of *in vivo* plaque microenvironment. This could be investigated by extending our model to include more microenvironmental factors and to account for changes in macrophage polarization in different microenvironmental mechanisms. For instance, there are many cytokines and chemokines involved in plaque progression and for several, an association with macrophage polarization has been shown [42–44]. Recent reports also suggest that collagen density in plaques may regulate macrophage polarization, which in turn affects MMP secretion and SMC proliferation and migration [11,12,45]. Our model could easily be extended to account for these effects, but at the cost of increasing the coupled PDEs and the number of estimated model parameters. Moreover, the sub-cellular pathophysiology, such as the relative signalling pathways and their influences on plaque cellular components, is able to be quantitatively analysed with the inclusion of ordinary differential equations into the proposed system. Ultimately, these models could be used to inform experimental design that in turn leads to improved model parameter estimations and validation of model predictions.

## 5. Conclusion

In order to investigate the mechanism of macrophage polarization in plaque progression, a dynamic stochastic mathematical model was established. The dynamic changes in plaque microenvironmental factors, including cellular and acellular components, were described by a series of PDEs, while the probability of macrophage phenotypic switching was described using a Bernoulli distribution. The simulation results presented the distribution of key microenvironmental factors, the dynamics of macrophage polarization and its influence on foam cell formation. In particular, M1 macrophages were found to predominate in advanced plaque corresponding to the exacerbated inflammation observed in mice experiments. In addition, the comparison of simulation results with experimental findings in targeted therapies indicated that the intervention and regulation of macrophage polarization could influence plaque microenvironment and subsequently induce plaque regression, especially in the early stage.

## Data Availability

Data are available from the Dryad Digital Repository: https://doi.org/10.5061/dryad.2280gb5v1 [46].
